# Plasmonic Hot Hole Generation by Interband Transition in Gold-Polyaniline

**DOI:** 10.1038/srep18276

**Published:** 2015-12-10

**Authors:** Tapan Barman, Amreen A. Hussain, Bikash Sharma, Arup R. Pal

**Affiliations:** 1 Plasma Nanotech Lab, Physical Sciences Division, Institute of Advanced Study in Science and Technology, Guwahati 781035, Assam, India

## Abstract

Studies on hot carrier science and technology associated with various types of nanostructures are dominating today’s nanotechnology research. Here we report a novel synthesis of polyaniline-gold (PAni-Au) nanocomposite thin films with gold nanostructures (AuNs) of desired shape and size uniformly incorporated in the polymer matrix. According to shape as well as size variation of AuNs, two tunable plasmonic UV-Visible absorption bands are observed in each of the nanocomposites. Plasmonic devices are fabricated using PAni-Au nanocomposite having different UV-Visible plasmon absorption bands. However, all the devices show strong photoelectrical responses in the blue region (400–500 nm) of the visible spectrum. The d-band to sp-band (d-sp) transition of electrons in AuNs produces hot holes that are the only carriers in the material responsible for photocurrent generation in the device. This work provides an experimental evidence of novel plasmonic hot hole generation process that was still a prediction.

The excellent light-trapping and local electromagnetic-field-enhancing properties of surface plasmons invite increasing research in the field of plasmonics thereby opening a wide range of applications in cancer therapy, photovoltaic devices, catalytic activity etc^1^,^2^,^3^. Under irradiation with matching frequency of the surface plasmon in well-designed nanostructures, localized surface plasmon resonance (LSPR) takes place resulting in intense local field enhancement^3^. Consequently, the plasmonic nanostructures are used as light trapping modules to enhance the performance of light harvesting devices^3^,^4^. Plasmonic photosensitization is another approach for achieving enhanced device performance beyond the spectral bandgap limit. The plasmonic hot carrier generation process is in the forefront of present research in many fields^1^,^2^,^3^,^4^,^5^,^6^. However, theoretical descriptions of hot carrier generation process are yet to be validated experimentally^7^. Reports are available on hot carrier generation due to the decay of localized surface plasmon in plasmonic nanostructures in n-type semiconductors, which mainly works on the principle of hot electron generation due to the decay of surface plasmon in the metal-dielectric interfaces^3^,^4^,^5^. There have been demonstrations of plasmonic oxidation driven by hot holes^8^,^9^. However, as per our literature survey no experimental evidence is available on any photovoltaic device that works on the principle of plasmonic hot hole generation process. Moreover, new predictions are coming, where it is claimed that plasmonic hot hole generation process in the p-type semiconductor is dominant, and the process could be more efficient than the plasmonic hot electron generation because the plasmon generated hot holes are ~2 eV more energetic than the plasmon generated hot electrons^7^.

Polyaniline (PAni) is a p-type organic semiconductor, widely synthesized by chemical or electrochemical wet methods that often involve a multistep processing, due to which there remains a limitation for its direct integration in organic devices^10^,^11^. Plasma polymerization along with simultaneous magnetron sputtering process emerges as a convenient method that meets all the criteria for preparing stable plasmonic material and its direct utilization in self-powered light harvesting devices^12^,^13^.

Here, we report the plasmonic hot hole generation due to the d-sp transition of AuNs in a PAni-Au nanocomposite based device configuration. The d-sp transition of electrons in AuNs that produces hot holes are the only carrier in the material that are responsible for photocurrent generation in the device.

The schematic of the present device is shown in Fig. 1a. The device structure comprises a PAni-Au nanocomposite layer sandwiched between a transparent indium tin oxide (ITO) anode and an aluminum cathode without any transport layer. The active material is successfully prepared at three different conditions (see Supplementary Information, Table S1). Based on the as-prepared PAni-Au nanocomposites, three different devices are fabricated namely, PD1, PD2, and PD3. The UV-Visible absorption spectra of PAni-Au nanocomposite prepared for PD1, PD2, and PD3 are shown in Fig. 1b. In all the three thin films, two peaks are present which are associated with the transverse and longitudinal plasmon resonances, and both the peaks get shifted depending upon the deposition conditions. The first peak is observed at 487 nm for PD1, 542 nm for PD2 and 595 nm for PD3 that is due to resonance of the transverse mode^14^,^15^,^16^,^17^. Similarly, resonance due to the longitudinal mode appears at 762 nm, 875 nm, and 956 nm for PD1, PD2 and PD3 respectively. Transmission electron microscopy (TEM) images of the PAni-Au nanocomposites for PD1, PD2 and PD3 are presented in Fig. 1c. Au nanorods are observed in PD1 and PD2 while random Au nanostructures are observed in PD3. It is found that the resonance frequency is strongly dependent on the aspect ratio of the nanorods which has values 2.8 and 1.4 for PD1 and PD2. The results obtained from TEM analysis are consistent with the presence of double absorption bands in the UV-Vis spectra.

The working principle of the present device is predictable from the energy level diagram presented in Fig. 2. The AuNs capture light of matching frequency of its surface plasmon resonance and energetic electron-hole pairs are generated due to the decay of plasmons^5^. The intraband transitions in the s-band of Au generate continuous energy distributions of electrons as well as holes extending from zero to the plasmon energy relative to the Fermi level^7^. These electrons cannot be injected since the maximum electron energy is nearly the plasmon energy above the Fermi level that is lower than the energy required to reach the lowest unoccupied molecular orbital (LUMO) of polyaniline (2.51 eV). Therefore, hot electrons generated in this process cannot reach the LUMO of PAni and the hot hole generated from the s-band electrons also unable to occupy the highest occupied molecular orbital (HOMO) of PAni. As a result, the excited s-band electrons cannot contribute to the photocurrent generation in the device.

It is well known that plasmon resonance in the d-band electrons also takes place in Au^7^. The energy of the d-band electrons lies ~2 eV below the Fermi level of Au^7^. Theoretically, from the *ab-initio* calculations, a peak in the joint density of states (JDOS) at a hole energy of ~2.7 eV is observed for Au^7^, which corresponds to the significantly higher number of hole generation under illumination of light at 2.8 eV^7^. The peak position does not change with the variation of incident light energy within the range of 2.6–2.8 eV. The electrons in the d-band may move to the sp-band with a d-sp transition absorbing light at a wavelength of ~450 nm (2.75 eV) since, the d-sp transition of Au occurs at an energy corresponding to this wavelength^14^,^15^. It is supported by the *ab-initio* calculations which reveal that in Au the allowed interband transition near the resonant surface plasmon polariton energies occurs at an energy within the range of 2.6–2.8 eV corresponding to 443–475 nm^7^. The decay of plasmons of the d-band electrons leaves holes which has energy below 2 eV than the Fermi level and are easily transferable to the HOMO of PAni due to favorable energy level alignment. Thus, barrier-less injection of plasmonic hot holes takes place from d-band of Au to HOMO of PAni^7^. If the prediction of this working principle is true, then all the three devices must work at the same wavelength (~450 nm) as the energy of d and sp-bands are material specific. Otherwise, the wavelength response of the device may vary with shape and size of the nanostructures as the plasmon absorption changes with shape and size.

The current-voltage (I-V) characteristics of the three devices (PD1, PD2, and PD3) are measured in the presence of light of different wavelengths (365, 450, 550, 650, 850 and 950 nm) at a fixed intensity of 2 mW/cm^2^ (Figure S1). The photoconductive gain *vs.* wavelength plot of the three devices are shown in Fig. 3a. Importantly, all the three devices show a maximum photoresponse at blue light (400–500 nm, λ_peak_ ~450 nm). It should be noted that, despite the significant shifting in the UV-Vis spectra, the devices do not respond to any other wavelength except ~450 nm. This observation directly points towards the contribution of plasmon generated hot holes, as proposed in the energy level diagram (Fig. 2.). For PD1, the transverse mode of plasmon resonance is close to the wavelength of blue light. Hence, PD1 shows a higher gain as compared to PD2 and PD3.

The I-V characteristics of the devices at dark and under irradiation of blue light at 2 mW/cm^2^ are presented in Fig. 3b. In the dark condition, all the three devices show a very low dark current of 1 × 10^−11^ A at 1.5 V bias. The current increases almost three order of magnitude with respect to the dark state under illumination at 0 V bias. PD1 shows better performance as compared to PD2 and PD3 because the surface plasmon resonance in PD1 takes place at 487 nm, which is close to the wavelength of d-sp transition. All the three devices exhibit photovoltaic properties, where PD1 shows maximum response with an open-circuit voltage (V_oc_) of 0.85 V and short-circuit current (I_sc_) of 5.12 nA respectively, which shows that the device could be useful in the self-powered mode as a photodetector. The photoconductive gain and detectivity of PD1 has values 0.0262 and 4 × 10^11^ Jones, respectively. The wavelength dependent parameters for PD1, PD2 and PD3 are summarized in Table S2.

The blue light intensity dependent I-V characteristics of PD1 is plotted in Fig. 3c. The current of the photodetector increases with increase in intensity. Intensity *vs.* photocurrent curve is shown in the Fig. 3d, where a non-linear behavior of the photocurrent is observed from the power law fit. The non-unity exponent is due to the various type of traps generated in the PAni^18^.

Figure 3e shows the photocurrent of PD1 during repetitive switching of blue light illumination at 0 V bias where the current increases 10^3^ times under illumination. The response time (τ_r_) of a photodetector is defined as the time required for increasing the photocurrent from 10 to 90% and the recovery time (τ_d_) is defined analogously. Figure 3f. Presents the single cycle on/off switching of the photodetector. From the first order exponential curve fitting of the single cycle on/off switching, the response time and recovery time constants of the device are calculated. The response time and recovery time constants of PD1 are found to be 16.02 ms and 17.12 ms, respectively. The results demonstrate the potential of applicability of the photodetector in optical switching devices or optical communication systems.

In summary, we highlight two important aspects of the present study. At first, an interesting physics of plasmonic hot hole generation by a d-sp transition in PAni-Au nanocomposite device is revealed. The work provides experimental evidence of novel plasmonic hot hole generation process. Secondly, we have shown that the mechanism of plasmonic hot hole generation is useful for the development of self-powered photodetector selective to blue light that corresponds to d-sp transition.

## Additional Information

**How to cite this article**: Barman, T. *et al*. Plasmonic Hot Hole Generation by Interband Transition in Gold-Polyaniline. *Sci. Rep.* **5**, 18276; doi: 10.1038/srep18276 (2015).

## Supplementary Material

Supplementary Information

## Figures and Tables

**Figure 1:**
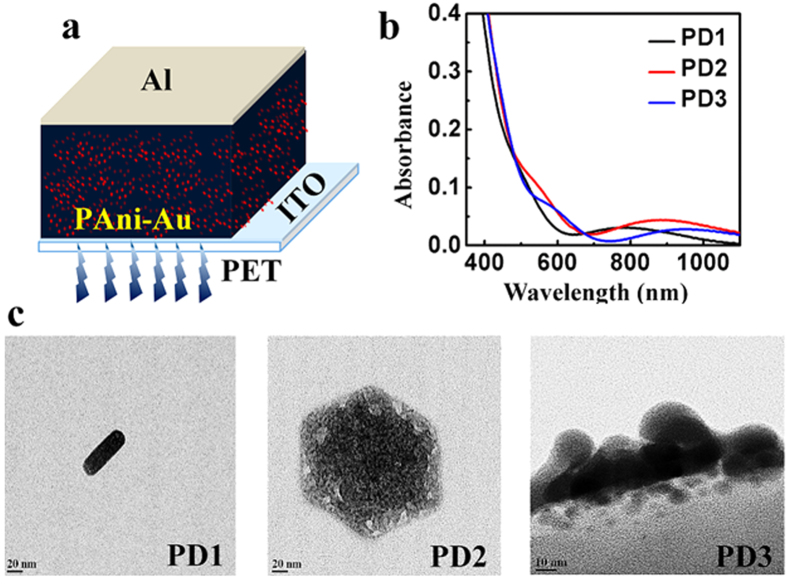
(**a**) Schematic illustration of the device, (**b**) UV-Vis absorption spectra of PAni-Au nanocomposite for PD1, PD2, and PD3, (**c**) TEM images of PAni-Au nanocomposite for PD1, PD2, and PD3.

**Figure 2:**
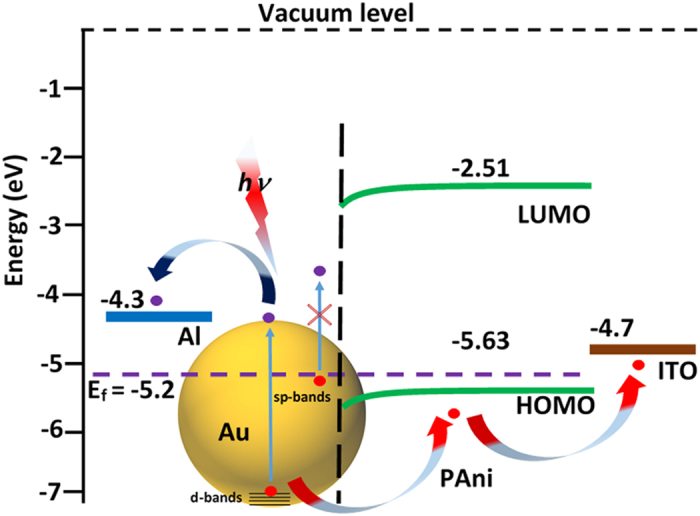
Energy level diagram and working principle of the device.

**Figure 3:**
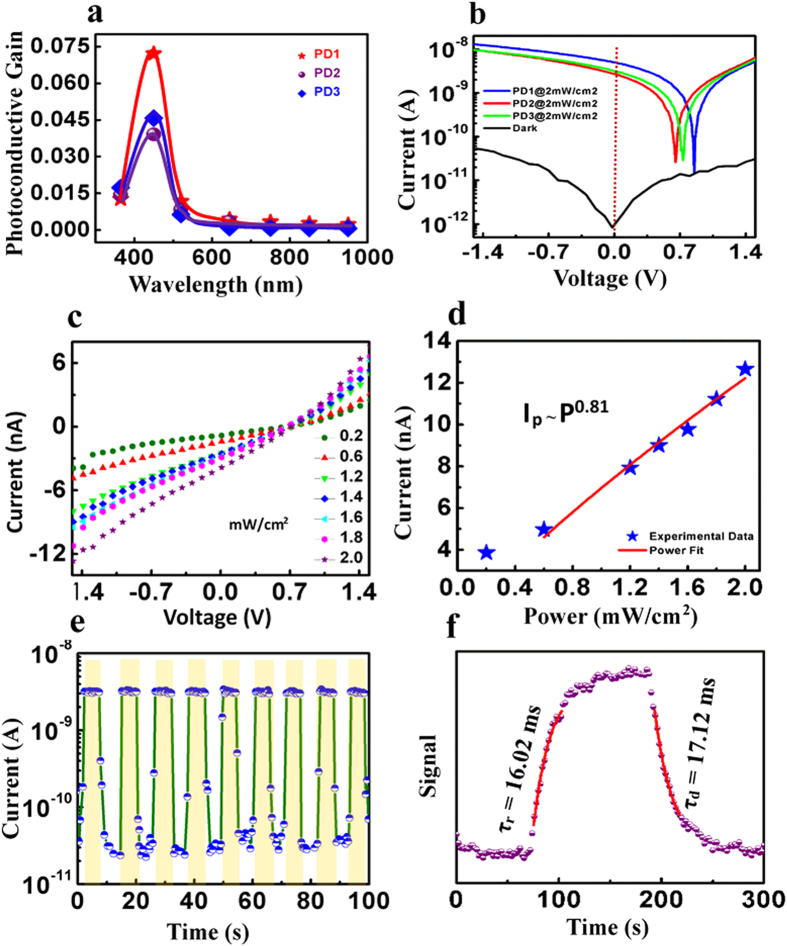
(**a**) Photoconductive gain *vs*. wavelength plot of PD1, PD2, and PD3, (**b**) dark current and photo current under illumination of blue light of intensity 2 mW/cm^2^ of the three devices, (**c**) intensity dependent I-V characteristic (**d**) corresponding power-law fit of PD1 under blue light, (**e**) Reproducible ON/OFF switching of the device under illumination of blue light at 2 mW/cm^2^, (**f**) Single cycle ON/OFF switching of the device.
