# Supplementary material for: Plasmonic Hot Hole Generation by Interband Transition in Gold-Polyaniline
Source: Sci Rep. 2015 Dec 10;5:18276. doi: 10.1038/srep18276 (PMC4674755; doi:10.1038/srep18276)
Supplement: Supplementary Information [file srep18276-s1.pdf]

Supplementary Information

Plasmonic Hot Hole Generation by Interband Transition in Gold-Polyaniline

*Tapan Barman, Amreen A. Hussain, Bikash Sharma, Arup R. Pal**

Plasma Nanotech Lab, Physical Sciences Division, Institute of Advanced Study in Science and Technology, Guwahati 781035, Assam, India

* To whom correspondence should be addressed. Tel.: +91 361 2912073; fax: +91 361 2279909; E-mail: arpal@iasst.gov.in (A. R. Pal)

Table S1. Device code and conditions of fabrication.

Device Code	Sputtering power (W)	Polymerization power (W)	Working Pressure (Torr)	Aniline flow (sccm)	Deposition time (min)	Thickness (nm)
PD1	35					418
PD2	55	10	5×10^{-2}	30	10	444
PD3	70					352

Figure S1. Wavelength dependent I-V characteristics of (a) PD1 (b) PD2 and (c) PD3 devices

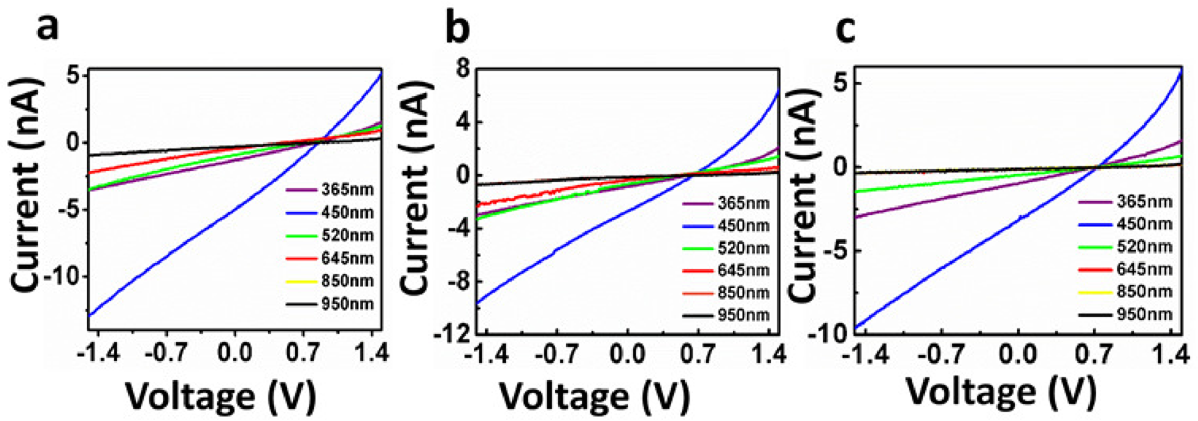

Table S2. Typical response parameters of the devices

Device	λ (nm)	P (mW/cm^2)	R_λ (A/W)	G
PD1	365	2	0.0067	0.0127
	**450**		**0.0262**	**0.0721**
	520		0.0049	0.0118
	645		0.0025	0.0045
	750		0.0023	0.0034
	850		0.0020	0.0028
	950		0.0019	0.0023
PD2	365		0.0042	0.0143
	**450**		**0.0142**	**0.0391**
	520		0.0035	0.0083
	645		0.0018	0.0035
	750		0.000759	0.0011
	850		0.000743	0.00096
	950		0.000733	0.00095
PD3	365		0.00510	0.0173
	**450**		**0.01660**	**0.0457**
	520		0.00270	0.0064
	645		0.00053	0.0010
	750		0.00055	0.000803
	850		0.00051	0.000695
	950		0.00053	0.000653

λ – wavelength, P – incident power density, R_λ – responsivity, G – photoconductive gain

Figure S2. **a.** Responsivity vs. wavelength plot of PD1, PD2, and PD3, **b.** External quantum efficacy (EQE) and internal quantum efficacy (IQE) of PD1 under illumination of blue light of intensity 2 mW/cm^2, **c.** variation of carriers collected per incident photon **d.** variation of carriers collected per absorbed photon.

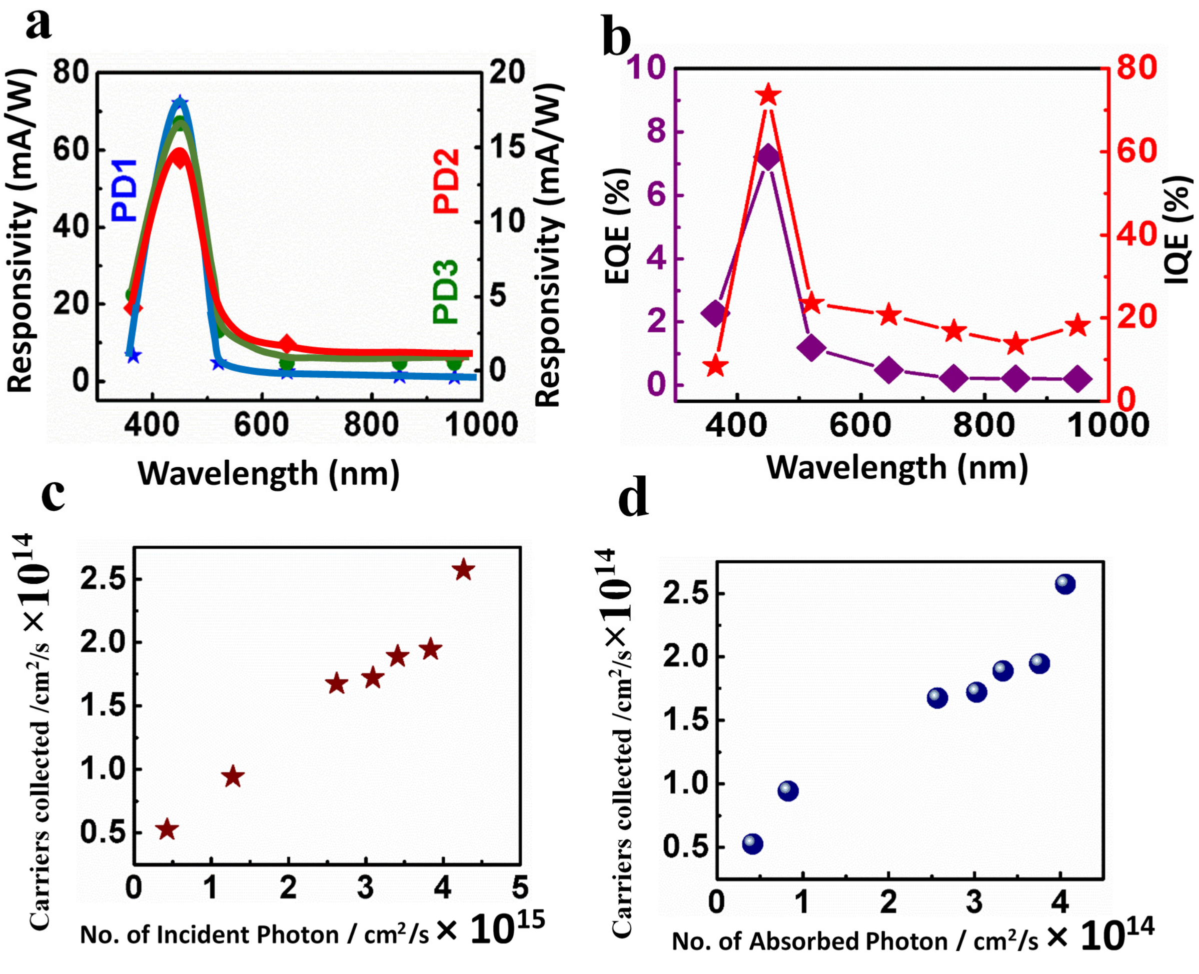

Experimental method

A stainless steel cylindrical plasma reactor of 40 cm in length and 30 cm in diameter is used in this work. The plasma reactor is equipped with a water cooled planner RF electrode of diameter 10 cm and a planner magnetron of diameter 2.54 cm fitted with a high purity (99.99%) gold target, maintaining separation of 10 cm[1-3]. The reactor is mechanically etched using fine polishing papers, chemically etched using acetone and then plasma etching is done at RF power of 100 W at a reactor pressure of 2×10^{-2} Torr in the presence of oxygen (10 sccm) and argon (30 sccm) for 45 min to remove the contamination. Before the introduction of any gas, the reactor is evacuated to a base pressure of 2×10^{-5} Torr by using a turbo molecular pump in combination with a dry roughing pump. Using a mass flow controller 40 sccm of pure argon (99.99%) gas is inserted into the reactor. By applying 50% speed of the turbo pump, argon pressure is maintained at 2×10^{-3} Torr. Aniline vapor is injected into the chamber at the partial pressure of 2×10^{-3} Torr. The flow of aniline vapor is controlled and recorded by a vapor source mass flow controller (1150, MKS Instruments, USA). Total reactor pressure with Argon and Aniline vapor is maintained at 5×10^{-2} Torr.

The magnetron system is connected to a pulse DC generator (Pinnacle Plus, Advanced Energy, USA) and power is adjusted to 35 W, 55 W, and 70 W at frequency 100 KHz and duty cycle 55%. The electrode assembly is connected to an RF generator (RF VII, USA), operated at 13.56 MHz and power is adjusted to 10 W. Chilled water (10° C) is allowed to pass through the magnetron and electrode assembly and both the power supplies for magnetron and electrode are switched ON. The deposition is carried out for 10 minutes. During polymerization, the reactor pressure is monitored by a capacitance manometer (Baratron, MKS Instruments, USA).

Device fabrication process

In this work we have fabricated 3 photodetectors namely PD1, PD2, and PD3, where PAni-Au nanocomposite thin film is the active material. The PAni-Au nanocomposite thin film is fabricated by plasma polymerization and simultaneous magnetron sputtering process. During the synthesis of PAni-Au nanocomposite thin films, the power applied in the magnetron is varied keeping the RF power fixed as listed in Table S1. The active material is sandwiched between transparent ITO coated PET and thermally evaporated Aluminum layers. The device has a lateral area of ~ 9.42×10^{-5} cm^2.

Thin film Characterization

The UV-Visible absorption spectra of the PAni-Au nanocomposite thin film deposited on the quartz substrate is recorded in UV3101PC, Shimadzu, Japan. Surface morphology of the PAni-Au nanocomposite thin film is recorded using a field emission scanning electron microscope (FESEM, ΣIGMA, Zeiss, Germany). The energy dispersive X-ray (EDX) spectra of the PAni-Au nanocomposite thin film deposited over a low doped silicon substrate is recorded using an FESEM coupled EDX. TEM analysis is carried out by JEM CM200 transmission electron microscope. The crystalline phase of PAni-Au nanocomposite is analyzed by X-Ray Diffractometer (XRD) (D8 Advanced, Germany) using Cu K_α radiation. HOMO and LUMO levels of PAni are determined from cyclic voltammetry[4,5].

Electrical measurements

The I-V characteristic of the present photodetectors are measured by Keithley 6517B electrometer/ high resistance meter in dark and under illumination of 365 nm, **450 nm,** 520 nm, 645 nm, 850 nm, and 950 nm LED light of intensity 2 mW/cm^2. For transient photocurrent measurement, the 450 nm light is switched ON and OFF for several times by using function generator at 0 V fixed bias, and a corresponding change in current is recorded.

Device parameter calculation

The measure of performance of photodetectors relies on the following important parameters (i) photoconductive gain (G), (ii) spectral responsivity (R_λ), (iii) specific detectivity (D*), and (iv) response speed.

The photoconductive gain of the photodetector represents the sensitivity to the signal. G is defined as the increase in number of collected carrier at the electrodes per unit absorbed photon (having energy hν) and mathematically expressed as[4];

$$G = \frac{\Delta I/q}{P/h\nu}$$

where, $\Delta I = I_{light} - I_{dark}$, q is the elementary charge and P is the incident light power.

Spectral responsivity (R_λ) is another important parameter that reflects the electrical output per optical input of a photodetector. R_λ is expressed as[4]:

$$R_\lambda = \frac{I_p}{PS}$$

Where, I_p is the photocurrent generated in the device, P is the incident power of the light signal, and S is the effective area of the device.

The detectivity determines the minimum strength of signal that can be detectable above the noise level and is expressed by formula[6]:

$$D = \frac{A^{1/2}R_\lambda}{(2qI_{dark})^{1/2}}$$

where, I_{dark} is the dark current density, q is the charge of the electron.

Figure S3. XRD pattern of PAni-Au nanocomposite.

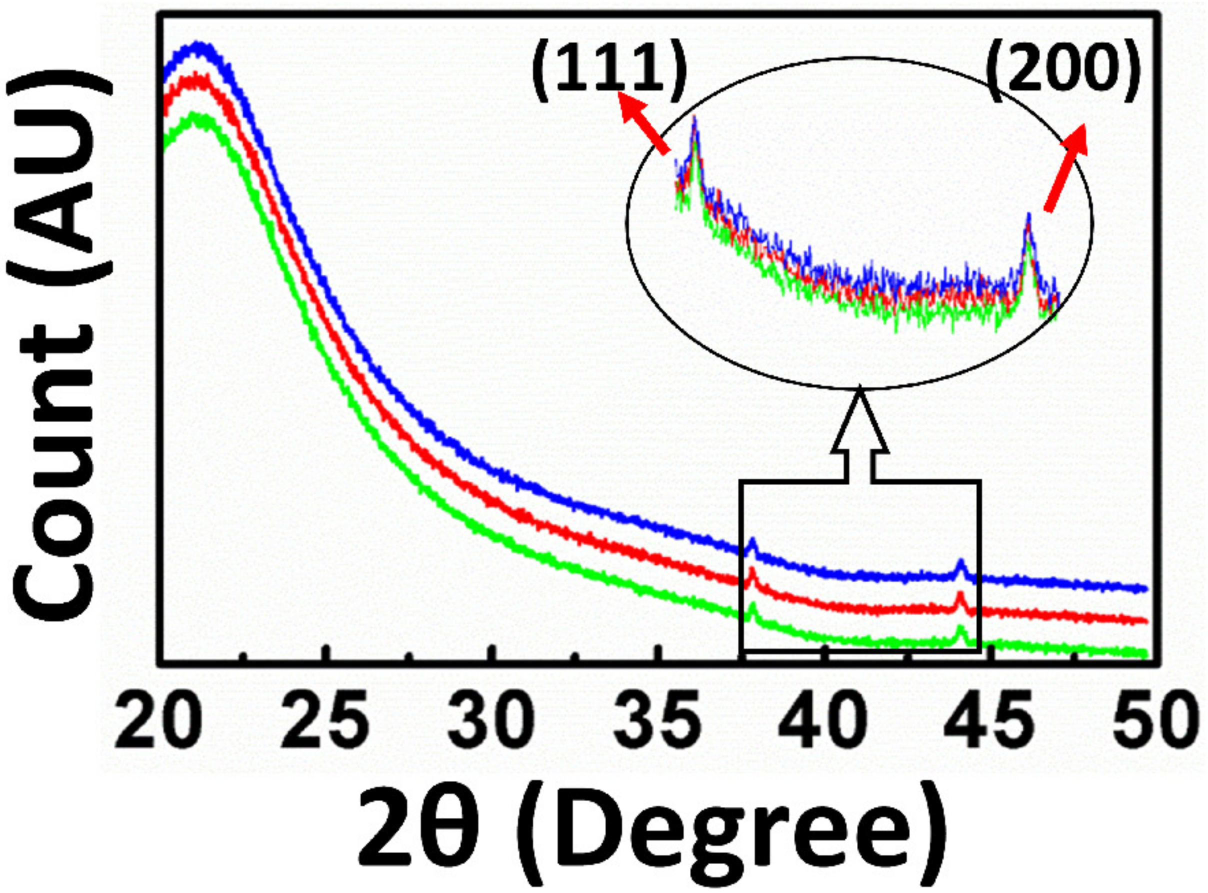

Figure S4. FESEM image of the PAni-Au nanocomposite film for (a) PD1, (b) PD2, and (c) PD3.

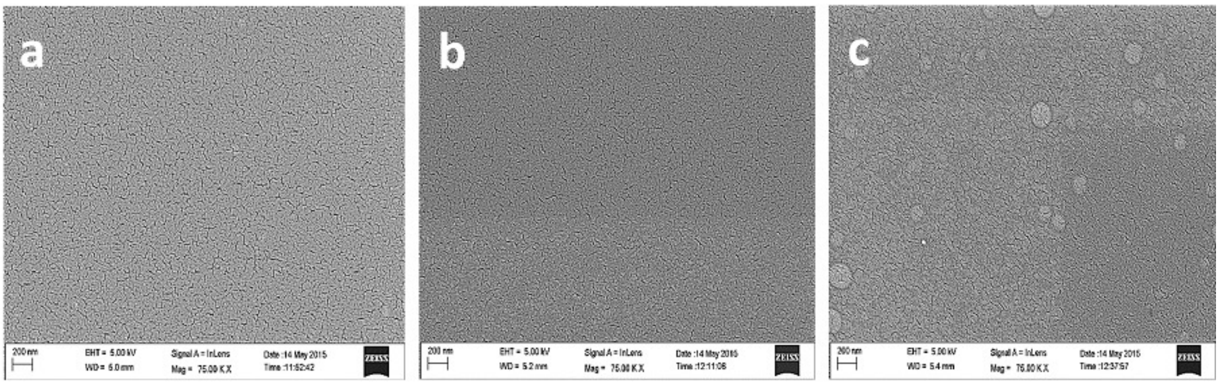

Figure S5. EDX spectrum and mapping of PAni-Au nanocomposite.

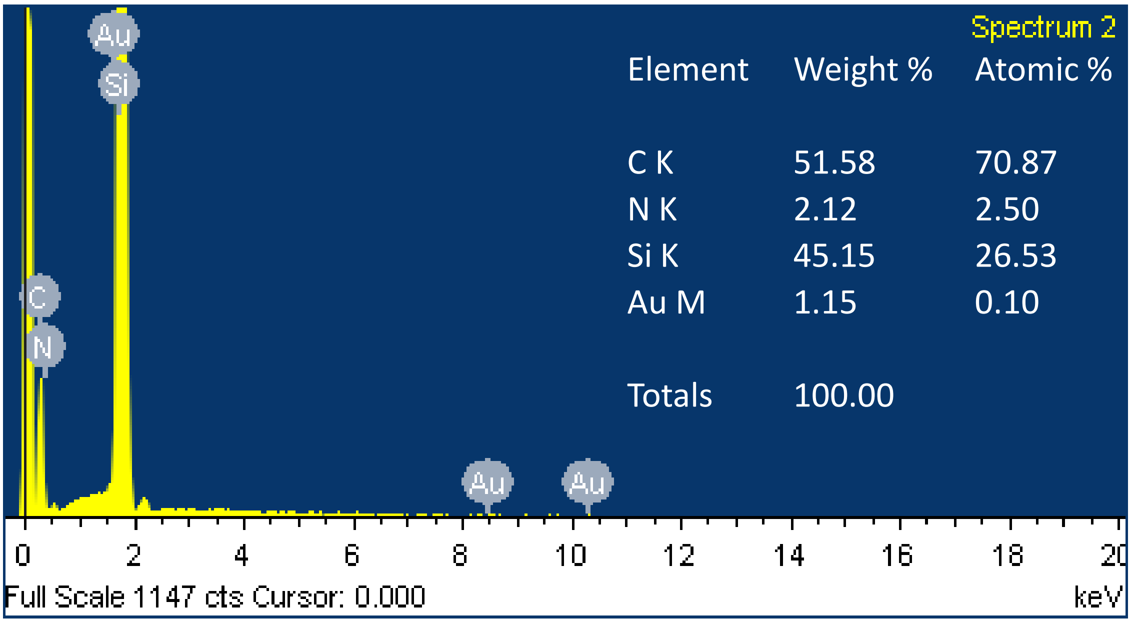

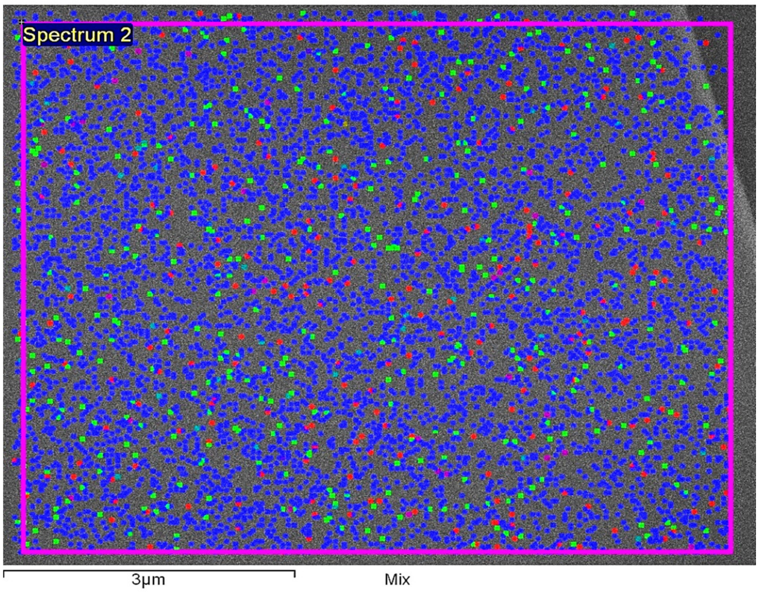

Figure S6. TEM morphologies of PAni-Au nanocomposite for (a) PD1 (b) PD2 and (c) PD3. The inset shows corresponding the SAED pattern.

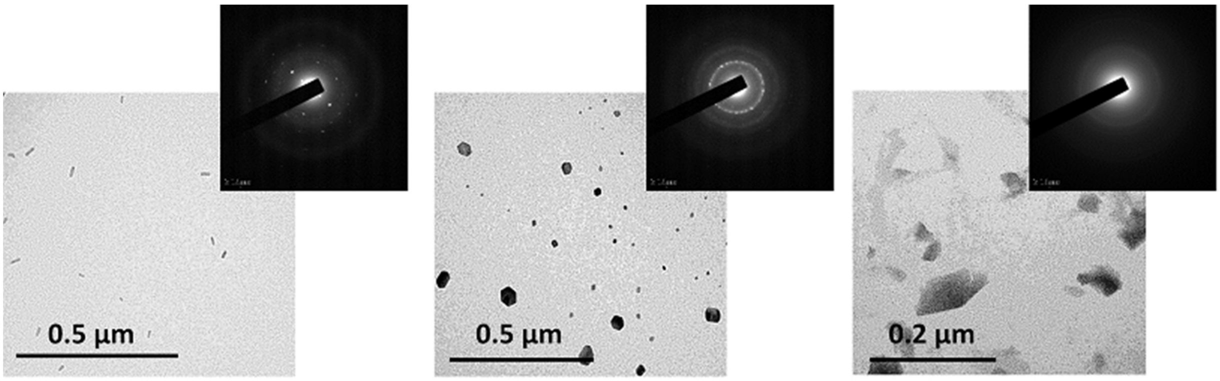

References

1. Barman, T., Pal, A. R. & Chutia, J. Comparative Study of Structural and Optical Properties of Pulsed and RF Plasma Polymerized Aniline Films. *Appl. Surf. Sci.* **313**, 286–292 (2014).

2. Agarwala, M. *et al.* Highly Effective Antibiofilm Coating of Silver–Polymer Nanocomposite on Polymeric Medical Devices Deposited by one Step Plasma Process *J. Biomed. Mater. Res., Part B* **102**, 1223-1235 (2014).

3. Gogoi, D. *et al.* Immobilization of Trypsin on Plasma Prepared Ag/PPAni Nanocomposite Film for Efficient Digestion of Protein. *Mater. Sci. Eng., C* **43**, 237–242 (2014).

4. Hussain, A. A., Pal, A. R. & Patil, D. S. High photosensitivity with enhanced photoelectrical contribution in hybrid nanocomposite flexible UV photodetector, *Org. Electron.* **15,** 2107–2115 (2014).

5. Hussain, A. A., Pal, A. R. & Patil, D. S. An efficient fast response and high-gain solar-blind flexible ultraviolet photodetector employing hybrid geometry, *Appl. Phys. Lett.* **104**, 193301 (2014)

6. Hong, Q. *et al.* Self-Powered Ultrafast Broadband Photodetector Based on p–n Heterojunctions of CuO/Si Nanowire Array, *ACS Appl. Mater. Interfaces* **6,** 20887–20894 (2014).
